# Unlocking sustainable livestock production potential in the Colombian Amazon through paddock division and gender inclusivity

**DOI:** 10.1038/s41598-024-63697-2

**Published:** 2024-06-13

**Authors:** Augusto Castro-Nunez, Alexander Buritica, Federico Holmann, Mary Ngaiwi, Marcela Quintero, Antonio Solarte, Carolina Gonzalez

**Affiliations:** 1 International Center for Tropical Agriculture - CIAT, Cali, Colombia; 2 The Nature Conservancy - Sustainable Productive Systems Program. TNC- Colombia CO, Bogota, Colombia; 3 Universidad de Los Andes, Bogota, Colombia

**Keywords:** Ecology, Environmental sciences, Environmental social sciences

## Abstract

Low-emissions livestock production can be achieved through scaling production systems integrating trees, forages, and livestock within the same area. Such systems are known as silvopastoral production systems (SPS). However, despite SPS reported benefits, adoption rates globally remain low. This paper, therefore, aims to inform land use policy oriented towards increasing SPS adoption. This study intends to capture this by assessing SPS adoption benefits, identifying determinants of SPS adoption, and thus to contribute to policy for scaling low-emissions livestock production. Data was collected on socioeconomic status, livestock farming technical and economic indicators, and farm and paddock practices through farm-level surveys in four municipalities in the Amazon Piedmont of Colombia. Unlike previous studies that assume homogeneous farm management, when in fact, it is heterogeneous, this study assessed SPS adoption determinants using the paddock (n = 2819) as the unit of analysis. This methodological approach is consistent with paddock-level land use decisions taken by farmers based on socioeconomic and biophysical factors such as soil financial resources, type, and topography. The methodological approach allows us to provide new insights into the determinant of adopting SPS and an understanding of intra-farm level land use decisions. The results show that the adoption of SPS at both paddocks and farm levels in Caquetá is low. The main factors associated with higher SPS adoption levels at the paddock level are framed in gender, resources, and knowledge. We observe that women are more motivated to conserve the environment. Cattle paddocks managed by women, smaller in size, and those with more SPS-related projects show a tendency for medium or high SPS adoption. Furthermore, the positive relationship between access to credit and SPS adoption emphasizes the importance of financial resources tailored to SPS projects. Enhancing gender roles, improving access to finance in land use, and providing training programs on SPS systems can contribute to low-emission livestock production in Colombia. This research paper highlights the significance of implementing diverse management strategies and reaching out to farmers not involved in SPS projects. It emphasizes investments in low-emission livestock production, especially for female heads of households. This approach recognizes the broader benefits of SPS, beyond production and financial gains, promoting the division of paddocks and the adoption of SPS.

## Introduction

The growing increase in demand for food of animal origin compels us to seek sustainable livestock production systems capable of mitigating the negative impacts associated with livestock farming^1^. Livestock production is associated with negative environmental externalities, including deforestation, biodiversity loss, and increased greenhouse gas (GHG) emissions^2,3^. In such context, silvopastoral production systems (SPS) are being promoted in various tropical countries as a means to reduce pressure on natural ecosystems, as well as to restore degraded land^4^. However, despite reported social and environmental benefits, the rate of SPS adoption globally remains low^5^.

SPS combine timber and fruit trees with pasture grasses and legumes to support animal grazing^6,7^. Such systems are especially beneficial when they include shrubs and trees with leaves edible for livestock, as these components are pivotal for the economic and sustainability success of SPS. They include various land management approaches, such as planting live fences with woody species (trees and shrubs), forage banks, scattered trees in pastures, grazing on plantations with timber or fruit trees, and windbreakers, among others^8,9^. SPS combines several agronomic practices and the implementation of shrubs and trees in different designs and densities with pasture species adapted to provide higher biomass yields and quality^10^. This is done in small land areas that seek better use of trees and forage diversity, more efficient use of resources, higher productivity, and a lower rate of natural landscape transformation^11–13^.

SPS are being promoted in both tropical and temperate countries as a pathway to contributing towards climate change mitigation. Particularly because of their potential to (1) increase carbon pools using livestock systems by planting trees; (2) increase carbon stocks in soil and pastures and shrubs biomass; and (3) reduce GHG emissions by increasing feeding efficiency in livestock systems^14^. Recently, it has been argued that SPS can also reduce pressure on forest resources if implemented alongside environmental safeguards to prevent potential deforestation leakages^15^. Likewise, evidence suggests that SPS increases the land value and productivity by diversifying and improving its production while sustainably managing soil resources to avoid fertility loss^4^. Consistently, countries with high losses of Amazon Forest areas, such as Colombia, have included SPS as part of Nationally Determined Contributions (NDC) submitted to the United Nations Framework Convention on Climate Change (UNFCCC).

Low SPS adoption rates in tropical countries have been attributed to the complexity of SPS innovations, the reluctance of farmers to invest and take risks with new technologies that require time before achieving profits, particularly in areas affected by armed conflict^16,17^. These adoption rates have also been ascribed to limited access to information and frequent technical assistance needed by SPS systems. Adding to the afore mentioned, few financial institutions have SPS options on their credit agendas, leading to inaccessibility or limited availability of appropriate financial and non-financial incentives to bolster SPS adoption^18^. In addition to these challenges, low understanding of social and gender dynamics can also play a significant role in the low adoption of SPS. For instance, research has shown that gender-related factors, such as differences in access to resources, decision-making power, and social norms, can influence farmers' adoption decisions^19–21^. Further, women who often have limited access to land, credit, and extension services, face additional barriers in adopting SPS practices^22^. This is because, in many agricultural communities, women are responsible for household food security and play a crucial role in managing natural resources^23^.

In most studies, SPS benefits have been assessed through ex-ante analysis or ex-post analysis performed at farm scale^18,24^. Likewise, SPS adoption studies usually use the farm as the unit of analysis. More specifically, these studies usually consider that a farmer is an adopter if SPS practices have been implemented in a portion, such as one paddock of the farm. By so doing, they only partially capture intra-farm level land use decisions. Consistently, most studies on the determinants of adoption of SPS have been performed mostly using the farm as the unit of analysis^25–28^. However, a better understanding of how intra-farm level land use decisions can affect the adoption of SPS would better inform the development of strategies and land use policies that increase SPS adoption rates.

The objective of this study was therefore to assess SPS adoption benefits, identifying determinants of SPS adoption, for informing efforts for scaling low-emissions livestock production. By so doing, this study will contribute to the understanding of intra-farm level land use decisions and how these decisions influence the adoption of SPS. Our analysis draws on data from 2819 paddocks across 213 livestock farms located in four municipalities within the Caquetá department of the Colombian Amazon. From our analysis, we advance from traditional farm-level analysis to paddocks level as the primary unit of analysis to better understand intra-farm level land use choices and how these choices influence the adoption of SPS. By doing this, this study points out the farm level benefits of SPS adoption and offers novel insights into the factors determining the paddock level adoption of SPS. These therefore explores the advantages tied to SPS adoption, and sheds light on the challenges, opportunities policy implications for the scaling of low-emissions livestock production systems in the Colombian.

## Livestock production in Caquetá Colombia

Caquetá is a hotspot for deforestation and extensive livestock production in Colombia. It is one of the departments most affected by deforestation due to factors such as land grabbing, cultivation of crops for illicit use and the expansion of grasslands for cattle ranching^29^. Furthermore, this department is also a priority region for implementing the peace agreement with the FARC guerrillas, a deal to end an internal conflict of more than fifty years of existence. Accordingly, national, and international cooperations are heavily investing in promoting sustainable livestock models and value chains in Caquetá to reduce the environmental impact of extensive cattle ranching, reduce deforestation, and strengthen the livelihoods of rural communities^15^. Consequently, promoting SPS as a sustainable alternative has always been at the core of investments in regions characterized by abundance of biological diversity and water resources, unsustainable natural resource exploitation and high presence of armed conflict and illicit crops^30^.

In Colombia, livestock production systems and grasslands have expanded from 14.6 to 24 million hectares (21% of the national area) in the last 50 years^31^. One historical cause for the growth of cattle farming is the governmental program to curb illicit coca cultivation, which encouraged the establishment of cattle farming as an alternative development program, allocating subsidies to farmers to enable them establish grasslands in areas used previously for illegal crops^32^. Livestock, therefore, plays an essential role in the economy of Colombia, by generating approximately 1.4% of Colombia’s Gross Domestic Product (GDP), 21.8% of the total agricultural GDP, and 19% of employment in rural areas^14^. It also contributes to the food supply for households, employment in rural areas, and is part of national poverty alleviation policies^33^. For small-scale farmers, livestock is an essential source of cash; it also supports agricultural diversification and farm investment^16,25^. Unfortunately, the livestock sector is the main driver of both deforestation and greenhouse gas (GHG) in Colombia and the department of Caquetá^34^.

## Methods

**Figure 1:**
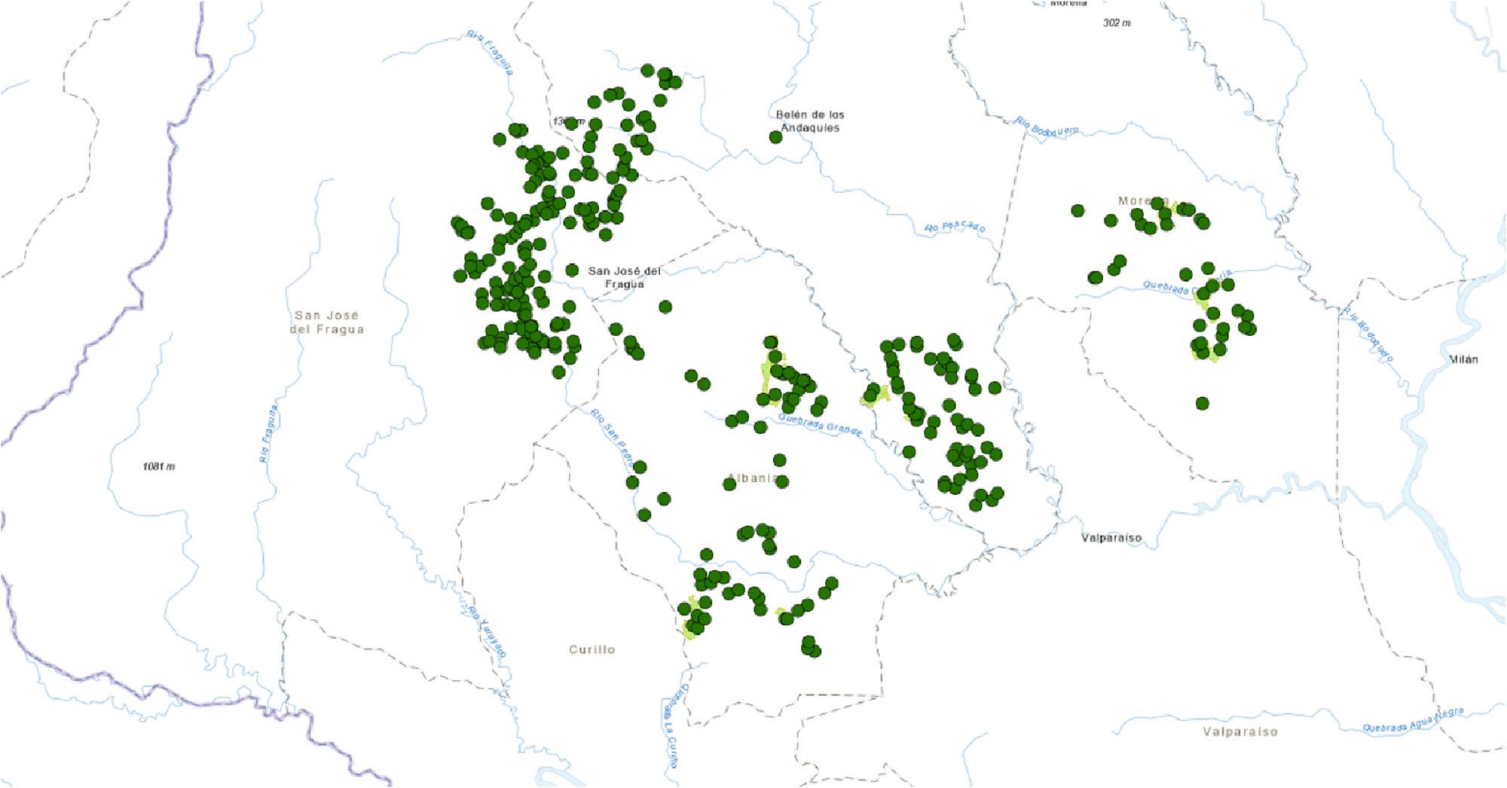
Map showing allocation of farms in study area-Caquetá, Colombia.( by A. Buritica using ArcGIS PRO version 3.1.0).

Socioeconomic data, including information from 2819 paddocks, was collected through farm level surveys within 213 farms of smallholder livestock producers during December 2019 and January 2020. As shown on Fig. 1, these surveys were carried out across four municipalities in the Caquetá department: Morelia (55 farms with 697 paddocks), Albania (71 farms with 878 paddocks), Belén de los Andaquíes (55 farms with 943 paddocks), and San José del Fragua (32 farms with 301 paddocks). Most of the interviews were conducted with the household head. When the household head was unavailable, the person in charge of agricultural activities on the farm was interviewed. The survey captured households’ and farms’ socioeconomic data, technical and economic indicators of livestock farming, number of paddock and paddock practices, and farmers’ reasons and conditions for adopting SPS practices.

To explore the factors associated with adoption of SPS and its effect on the productive variables of cattle farms we appliedthe following steps. First, based on experts’ opinions we classified both paddocks and farms into levels of SPS adoption. Second, we used descriptive statistical analyses to provide an overview of the characteristics of paddocks and farms as well as to assess the farm level benefits of adopting SPS. To accomplish this, we conducted a t-test to compare farms based on their classification according to the levels of SPS adoption in their paddocks. This analysis was carried out at the paddock level using farm-level productive variables, as there are no available measurements specifically at the paddock level for productivity.

Lastly, we developed an econometric multinomial logit model to identify the determinants of adoption of SPS using the paddock as the unit of analysis. In this econometric model, we integrate socio-economic variables that evaluate the characteristics of the farm, the head of the household, and the paddocks, offering valuable insights into the factors that impact the adoption of SPS practices. Finally, based on the results obtained from the multinomial logit model, the predicted probability of a paddock belonging to each SPS adoption category for each continuous variable used in the econometric model is calculated and plotted. Based on this, a descriptive analysis was made of the relationship between each variable and the probability of belonging to each SPS category adoption.

### Definition of adopters

This study establishes a criterion based on experts’ opinions to classify paddocks (a defined and enclosed area of land used for agricultural purposes, typically for grazing livestock or growing crops) into four levels of SPS adoption. According to the practices implemented in cattle paddocks, two groups were considered as non-adopters and two as adopters. These categories represent a continuum along SPS adoption, from less-intensive and less-expensive SPS to a more complex system that involves more investment (in planted trees, wire fencing, and improved pastures). We refer to these categories as:

- *Non-adopter paddock (A1)* Paddocks with only natural pastures. Does not carry out any practice or a combination of practices that do not qualify as SPS practices.
- *Non-adopter with improved pasture paddock (A2)* Paddocks with natural and improved pastures.
- *Adopter-basic paddock (A3)* Paddocks with natural, improved pastures and planted trees (higher percentage) and stable trees.
- *Adopter-medium–high paddock (A4)* Paddocks with natural, improved pastures, planted and stable trees (higher percentage), with livestock aqueduct and fodder bank.

Since a farm has multiple paddocks, the same farm may have paddocks that do not adopt SPS (A1 or A2) and paddocks with the highest levels of adoption of SPS (A3 or A4). Therefore, we also classify farms into four SPS adoption categories defined based on the paddock’s classification, which have the following categories:

- *Non-adopter farm (FA1)* Farms with paddocks only with natural pastures (paddocks A1 type).
- *Non-adopter with improved pasture farm (FA2)* Farms with paddocks with natural or improved pastures (paddocks A1 and A2 type).
- *Adopter-basic farm (FA3)* Farm with paddocks with natural and improved pastures and planted and stable trees (paddocks A1, A2, and A3 types).
- *Adopter-medium–high farm (FA4)* Farm with paddocks on its farm with different levels of SPS adoption. These farms may have non-adopting paddocks, such as with some SPS adoption (paddocks A1, A2, A3, and A4 types).

### Empirical strategy

We used two empirical approaches: (i) descriptive statistics was used to characterize and classify paddocks and farms according to SPS adoption intensity and observe the effect of adoption on productive variables at farm level; and (ii) a quantitative strategy used to understand the factors associated with SPS adoption through a Multinomial Logistic model at paddock level. The multinomial logistic model was used because it makes it possible to predict the probability that an individual/agent belongs to some category. In this case, we use the model to predict the likelihood that a paddock belongs to a mutually exclusive adoption level. Specifically, we propose the following equation:

$${\text{p}}^{{\text{j}}} = \frac{{\exp \;\left( {X^{\prime}\beta_{{\text{j}}} } \right)}}{{1 + {\text{D}}}}$$

where ${\text{p}}_{\text{j}}$ is the probability that a paddock is at adoption level $\text{j}$.

$\text{j}=(\text{1,2},...,\text{m}-1)$ are the adoption levels.

$\text{D}$ is the choice probability of the alternative with ${\text{D}} = \mathop \sum \limits_{{{\text{j}} = 1}}^{{{\text{m}} - 1}} \exp \left( {X^{\prime}\beta_{{\text{k}}} } \right)$.

k=(1,2,…m).

$\text{X}$ Is a vector of characteristics at the paddock and farm level, and ${\upbeta }_{\text{j}}$ is the coefficient vector belonging to adoption level $\text{j}$. The vector X comprises farm-level variables, including factors like the age of the head of the household, access to credit, herd size, whether the household has previously benefited from an SPS project, and awareness of existing SPS cases within its municipality. Table 1 depicts an explanation of variables used in this study. Additionally, at the paddock level, we account for their size in hectares.

**Table 1:** Description of variables.

Variables	Calibration	Expected sign
Age	Age of farm house head (Years)	±
Gender	Gender of farmhouse head (Dummy, Female = 1 and Male = 0)	±
Cattle paddocks	Cattle paddocks in farm (Number)	±
Farm size	Farmland size (in Hectares (Ha))	±
Herd size	Animals in a Herd (Number)	±
Number of SPS projects	Number of SPS projects in the area (Number)	±
Credit access	Access to agricultural Credit (Dummy,Yes = 1, No = 0)	+
SPS success stories	Number of SPS success stories in the area (Number)	±
Multiple practices	Use of different practices on paddock (Yes = 1, No = 0)	+
Conservation agreements	Farmers participation in conservation agreements (Dummy, Yes = 1, No = 0)	+
Paddock size (Ha)	Paddockland size (Hectares)	±
Stocking rate	Number of animals grazing on a given amount of land for a specified time (Number)	±
Calving rate	The number of calves actually produced by a cow divided by the number of potential calves (%)	±
Milk productivity	Number of liters of milk produced by a cow per day (Lt/cow/day)	±
Income from sales of animals	Amount of animal sales in Colombian peso per year (Million COP/yr)	±
Income from milk sales	Amount of milk sales in Colombian peso per year (Million COP/yr)	±

## Results

### Social and economic statistics of Paddock and farms

Table 2 presents an overview of the paddock’s characteristics. On average, the age of the household heads is 52.18 years, with a standard deviation of 13.42. Additionally, only 15% of the sampled farms had female household heads.

**Table 2:** Descriptive statistics for farm and paddock variables.

	N	Mean	S.D	Min	Max
Farm Level variables (N = 190)					
Age of HH (Number)	2745	52.18	13.42	22	92
Gender (Female = 1 and Male = 0) ^λ^	2745	0.15	0.34	0	1
Cattle paddocks (Number)	2814	26.61	18.38	1	80
Farm size (Ha)	2819	45.03	27.78	0.5	134.5
Herd size (Number)	2786	50.29	30.27	1	136
Number of SPS projects	2819	0.64	0.72	0	3
Credit access (Yes = 1, No = 0)^λ^	2709	0.56	0.50	0	1
SPS success stories^δ^	2739	0.45	1.84	-9	8
Use of different practices on paddock (Yes = 1, No = 0)^γ^	2815	0.27	0.44	0	1
Conservation agreements (Yes = 1, No = 0) ^λ^	2617	0.42	0.49	0	1
Paddock Level variables (N = 2819)					
Paddock size (Ha)	2141	1.77	1.66	0.2	20

^λ^The estimate is interpreted as a percentage in relation to yes responses “Yes = 1”.

^δ^This variable was created as the difference between two questions: How many SPS success stories do you know in your municipality, and how many SPS success stories do you know in other municipalities? The positive values of the variable indicate that they know more success stories in their municipality than in the rest of the region.

^γ^This variable is a dummy variable that takes the value of 1 when the farmer employs distinct management practices in the various paddocks of their farm. In other words, it indicates whether the farmer implements different approaches or strategies for managing different areas within their farm.

On average, a farmer from the sample area has benefited from SPS projects at least 0.64 times. However, the high standard deviation in this variable (0.72) indicates that projects have concentrated only on some farmers and not evenly distributed among farmers (see Appendix A.1). Our results indicate that 56% of farmers in the studied population have access to credit, and 42% have engaged in a conservation agreement on their farms. On the other hand, the results indicate that, on average, cattle farmers know at least one more successful story of silvopastoral systems (0.45) in their municipality compared to other regional municipalities. Moreso, a standard deviation of 1.84 tells us that the number of silvopastoral systems success stories known by farming households tends to vary modestly, indicatinga relatively consistent level of awareness among the surveyed households. Furthermore, 27% of the farmers reported to have used at least 1 SPS practice on their paddocks.

Results from this study showed that 75.81% of the paddocks did not adopt sustainable livestock practices (Table 3). Specifically, from a total of 2819 paddocks, 34% were in the category of non-adopters, 42% in non-adopters with improved pasture, 17% adopter basic, while 7% adopter medium high level. Table 3 shows the distribution of the total number of cattle paddocks according to the levels of adoption.

**Table 3:** Paddock classification according to SPS adoption level.

SPS Type-Paddock	Freq	Percent	Cum
Non-adopter (A1)	952	33.77	33.77
Non-adopter with improved pasture (A2)	1185	42.04	75.81
Adopter-basic (A3)	488	17.31	93.12
Adopter-medium–high (A4)	194	6.88	100
Total	2819	100	

Descriptive statistics on paddock and farm classification according to SPS adoption level is depicted on Table 4 showing the number of farms, the average count of paddocks, the mean count of paddocks based on SPS adoption levels, the average sizes of farms and paddocks, as well as their engagement in conservation agreements for each of the previously mentioned categories at the farm level. The results indicate that there is a range of adoption levels of sustainable pastoral systems (SPS) within cattle farms, which is influenced by the unique characteristics of their paddocks. Specifically, the findings show that around 33% of the farms can be classified as adopters (Farms FA3 and FA4). Approximately 28% of the sample has paddocks type A3 and 5% has paddocks type A4. Within this last group of farms, 10 had implemented Sustainable Production Systems (SPS) across most of their paddocks. Farms that adopted SPS to a greater extent in their paddocks also showed higher levels of involvement in conservation agreements and had larger numbers of smaller paddocks. In other words, as farms implement SPS practices more comprehensively, they are more likely to participate in conservation agreements.

**Table 4:** Farm classification according to SPS adoption level.

Farm level (N = 190)	N	Mean	S.D	Min	Max
Adoption Category_Farm-FA1 (N = 43)					
Cattle paddocks (Number)	43	8	6	1	26
Cattle paddocks-SPS Type A1 (Number)	43	8	0	8	8
Cattle paddocks-SPS Type A2 (Number)	43	0	0	0	0
Cattle paddocks-SPS Type A3 (Number)	43	0	0	0	0
Cattle paddocks-SPS Type A4 (Number)	43	0	0	0	0
Paddock size (Ha)	43	3.20	2.46	0.33	10.25
Farm size (Ha)	43	29.34	19.33	2.4	78
Conservation agreements (Yes = 1, No = 0) ^λ^	40	0.13	0.33	0	1
Adoption Category_Farm-FA2 (N = 84)					
Cattle paddocks (Number)	84	19	15	3	80
Cattle paddocks-SPS Type A1 (Number)	84	5	8	0	18
Cattle paddocks-SPS Type A2 (Number)	84	14	8	0	19
Cattle paddocks-SPS Type A3 (Number)	84	0	0	0	0
Cattle paddocks-SPS Type A4 (Number)	84	0	0	0	0
Paddock size (Ha)	84	2.03	1.60	0.25	10.00
Farm size (Ha)	84	43.78	27.98	4	134.5
Conservation agreements (Yes = 1, No = 0) ^λ^	81	0.27	0.45	0	1
Adoption Category_Farm-FA3 (N = 53)					
Cattle paddocks (Number)	53	14	10	1	35
Cattle paddocks-SPS Type A1 (Number)	53	4	6	0	14
Cattle paddocks-SPS Type A2 (Number)	53	0	1	0	3
Cattle paddocks-SPS Type A3 (Number)	53	10	6	0	14
Cattle paddocks-SPS Type A4 (Number)	53	0	0	0	0
Paddock size (Ha)	53	2.31	2.61	0.20	18.00
Farm size (Ha)	53	31.50	25.51	2.5	123.5
Conservation agreements (Yes = 1, No = 0) ^λ^	49	0.47	0.50	0	1
Adoption Category_Farm-FA4 (N = 10)					
Cattle paddocks (Number)	10	23	12	2	40
Cattle paddocks-SPS Type A1 (Number)	10	2	7	0	22
Cattle paddocks-SPS Type A2 (Number)	10	0	0	0	0
Cattle paddocks-SPS Type A3 (Number)	10	0	0	0	0
Cattle paddocks-SPS Type A4 (Number)	10	21	7	1	23
Paddock size (Ha) ^δ^	9	1.77	1.15	0.25	3.50
Farm size (Ha) ^δ^	9	40.17	20.29	17	71.5
Conservation agreements (Yes = 1, No = 0) ^λ^	10	0.80	0.42	0	1

^δ^A single farm exhibited outliers’ values and was consequently excluded from the analysis.

^λ^The estimate is interpreted as a percentage in relation to yes responses “Yes = 1”.

More specifically, we identified 43 farms falling into the non-adopter category (FA1). These farms, on average, had 8 paddocks with a mean size of 3.20 hectares. Further, a significant number of farms (84) were classified as non-adopters with improved pasture (FA2). These farms, on average, comprised of 19 paddocks, each averaging 2.03 hectares in size. Moreover, 53 farms were categorized in the basic adopter category (FA3), encompassing an average of 14 paddocks with a mean size of 2.31 hectares (Table 4). Finally, farms classified as adopter-medium–high (FA4) had an average of 23 paddocks, each with a mean size of 1.77 hectares. Interestingly, within this latter group, only 2 out of the 23 paddocks were non-adopters, while 21 exhibited medium–high adoption levels. These results suggest that the adoption of SPS follows a gradual, long-term trajectory, necessitating not only access to information and resources, but also sufficient time for on-farm experimentation and a steadfast commitment to conservation practices.

### Farm-level benefits of Implementing SPS in Paddocks

Results in Table 5 depict that, the non-Adopters in Category Farm (FA1) have the highest mean stocking rate of 1.57 AU/Ha, but also high variability standard deviation (SD = 2.10), indicating less uniformity in stocking density compared to other groups. Additionally, the calving rates for the four fields analysed were as follows: FA1 had a mean rate of 58.63% (SD = 34.84), FA2 had a mean rate of 60.58% (SD = 20.05), FA3 had a mean rate of 61.28% (SD = 26.15), and FA4 had a mean rate of 67.02% (SD = 21.37). Thus, FA4 farms show the highest average calving rate and more consistent outcomes than FA1, which has a similar mean but a higher SD. Furthermore, milk productivity, measured in liters per cow per day, varied across four feeding areas (FAs) as follows: FA1 has a mean of 3.95 L (SD = 1.81), FA2 shows a mean of 3.94 L (SD = 2.09), FA3 presents a mean of 3.65 L (SD = 1.86), and FA4 records a mean of 3.15 L (SD = 1.43). These suggest that milk productivity is relatively similar across all farm types, though FA1 and FA2 have slightly higher means than FA3 and FA4. However, FA4 shows the most consistent productivity, evidenced by the lowest SD (see Appendix A.2).

**Table 5:** Descriptive statistics of productive farm variables by level of SPS Adoption.

Farm level (N = 190)	N	Mean	S.D	Min	Max
Adoption Category Farm-FA1 (N = 43)					
Stocking rate (AU/Ha)	38	1.57	2.10	0.00	10.50
Calving rate (%)	34	58.63	34.84	0.00	100.00
Milk productivity (Lt/cow/day)	17	3.95	1.81	2.22	8.57
Income from sales of animals (million COP/yr)	6	2.18	0.87	0.90	3.56
Income from milk sales (million COP/yr)	15	10.03	5.80	4.38	23.00
Adoption Category Farm-FA2 (N = 84)					
Stocking rate (AU/Ha)	83	1.09	0.76	0.00	4.70
Calving rate (%)	81	60.58	20.05	0.00	100.00
Milk productivity (Lt/cow/day)	76	3.94	2.09	1.33	17.62
Income from sales of animals (million COP/yr)	28	6.92	8.84	0.80	42.21
Income from milk sales (million COP/yr)	72	18.00	17.50	1.80	131.40
Adoption Category Farm-FA3 (N = 53)					
Stocking rate (AU/Ha)	50	1.44	1.24	0.30	7.75
Calving rate (%)	49	61.28	26.15	0.00	100.00
Milk productivity (Lt/cow/day)	43	3.65	1.86	0.83	10.00
Income from sales of animals (million COP/yr)	17	4.28	7.95	0.50	34.50
Income from milk sales (million COP/yr)	41	17.26	11.36	1.83	45.99
Adoption Category_Farm-FA4 (N = 10)					
Stocking rate (AU/Ha)	10	1.32	0.75	0.65	2.87
Calving rate (%)	10	67.02	21.37	25.00	100.00
Milk productivity (Lt/cow/day)	9	3.15	1.43	1.67	5.50
Income from sales of animals (million COP/yr)	7	4.57	6.37	1.00	18.90
Income from milk sales (million COP/yr)	8	15.08	6.53	7.80	26.10

Table 5 further shows that, the annual income from sales of animals, expressed in millions of Colombian Pesos (COP), varies across the four different farm categories, with FA1 having a mean income of 2.18 million COP (SD = 0.87), FA2 showing a mean of 6.92 million COP (SD = 8.84), FA3 having a mean of 4.28 million COP (SD = 7.95), and FA4 with a mean of 4.57 million COP (SD = 6.37) However, FA2 farms report the highest average income from animal sales, but also the highest variability. FA1 has the most stable income from this source, with the lowest SD. The annual income from milk sales, measured in millions of Colombian Pesos (COP), varied across different farm categories (Table 5) with FA1 reporting a mean of 10.03 million COP (SD = 5.80), FA2 showing a mean of 18.00 million COP (SD = 17.50), FA3 with a mean of 17.26 million COP (SD = 11.36), and FA4 having a mean of 15.08 million COP (SD = 6.53). FA2 again shows the highest mean income from milk sales, but with a substantial SD, indicating significant variability in income. In contrast, FA4 displays a more moderate mean with relatively low SD, suggesting a more consistent revenue from milk sales across these farms.

These results thus imply that, farms with higher levels of adoption (FA3, FA4) tend to show more consistency in their outputs and revenues, which can be inferred from their generally lower SDs compared to the high variability seen in the non-adopter and non-adopter with improved pasture categories (FA1, FA2). This suggests that adoption of improved practices and diversification in paddock usage might contribute to more predictable and possibly more efficient farm operations.

### Determinants of adoption of Silvopastoral Systems at Paddock level

Results frommultinomial logistic regression (MNL) analysis conducted using Stata version 18, in Table 6. MNL regressions were estimated for the entire study sample who adopted different categories of silvopastoral practices adoption at paddock level. The dependent variable consists of four options including ‘non-Adopter (A1) non-adopter with improved pasture (A2), Basic Adopters (A3) to Medium -High Adopters (A4)’. The final model exhibited robustness following a sequence of post-estimation assessments. From these estimations, we found no statistically significant Likelihood ratio tests for independent variables (Ho: B = 0) or Wald tests for simple or composite linear hypotheses of individual parameters. The Small-Hsiao test for assessing the IIA assumption did not yield significant results. Standard errors derived from the Huber-White sandwich estimator did not yield markedly divergent outcomes furthermore,regression diagnostics did not reveal any observations exerting undue influence on the results.

**Table 6:** Multinomial Logit model results at paddock level.

Variable	Multinomial logit model- marginal effects			
	Non-adopter(A1)	Non-adopter with improved pasture (A2)	Basic adopt (A3)	Medium-high adopt (A4)
Age of household head	− 4.40e−05	− 0.00182*	0.00254**	− 0.000676
	(0.000401)	(0.000855)	(0.000798)	(0.000481)
Gender of household head	− 0.0865***	− 0.503***	0.393**	0.197
	(0.0190)	(0.0110)	(0.140)	(0.139)
Age of household head × Sex of household head	− 0.000814	0.0260***	− 0.0184***	− 0.00682***
	(0.00304)	(0.00299)	(0.00279)	(0.00103)
Paddock size (Ha)	0.0409***	− 0.0343***	0.0216**	− 0.0282**
	(0.00426)	(0.0104)	(0.00794)	(0.00974)
Total herd size (number)	− 0.00332***	0.00392***	− 0.00134**	0.000742***
	(0.000350)	(0.000434)	(0.000450)	(0.000184)
SPS projects interventions	0.0763***	− 0.237***	0.0847***	0.0763***
	(0.0111)	(0.0183)	(0.0170)	(0.00962)
SPS success stories	− 0.00305	− 0.0443***	0.0352***	0.0122***
	(0.00270)	(0.00634)	(0.00553)	(0.00334)
Credit access	− 0.0212	0.126***	− 0.189***	0.0842***
	(0.0142)	(0.0214)	(0.0215)	(0.0144)
Observations (N)	2028	2028	2028	2028

Standard error in parentheses**.** *** p < 0.01; ** p < 0.05; *p < 0.1.

Table 6 presents the marginal effects derived from the multinomial logit model, giving insights on the diverse categories of SPS adoption at the paddock level. This methodology significantly enhances our comprehension of the SPS adoption process by shedding light on the distinct influence of each variable within different paddock categories, all based on the available data.

The results indicate that there is a significant positive relationship between paddock size and SPS project interventions with the Non adopter (A1) category. Additionally, the coefficients for gender and total herd size of paddock were found to be significant and negative. The negative correlation with gender suggests that female farmers have a lower likelihood of keeping the non-silvopastoral system compared to their male counterparts. Conversely, the negative association with total herd size implies that farmers with larger herds are less inclined to keep the non-silvopastoral system. Further, our results further indicate that age of household head, gender, paddock size, SPS project interventions, and number of success stories were significant negatively related to the non-adopter with improved pasture (A2) category. However, the relationship between total herd size and access to credit with the same category was positively significant.

There was a significant and positive association between total herd size and access to credit with the non-adopter with improved pasture (A2) category in the multinomial logit regression results. Implying that farmers with increased herd size an access to credit by one unit are more likely to be a non-adopter but with improved pasture. On the adopters’ side, age of the head of household and access to credit were negatively associated with the basic adopter paddock category (A3), at 1% significance level. Also, a negative relationship was observed with the cattle herd size with a statistical significance at 5%. Nonetheless, age of household head, gender, and paddock size, were observed to positively influence farmers to adopt SPS on paddocks with A3 category at 5% significance level. Meanwhile, PS project intervention and SPS success stories positively and strongly influence adoption at category A3 at 1%. This implies that as the number of SPS project interventions at farm level increases the farmer is more likely to adopt SPS on paddock with A3 category. Also, as the farmer knows of more SPS success stories from their municipality there is a high likelihood for them to adopt SPS on paddocks of category A3.

The herd size, SPS project intervention, SPS success stories and access to credit are strongly and positively associated with medium–high adopter paddocks (A4). A one-unit increase in SPS projects and SPS success stories elevates the likelihood of a farmer adopting SPS on a medium–high adopter paddock. furthermore, medium–high adopter paddocks (A4) are positively associated with access to credit, which implies that a one unit increase in credit access increases the likelihood for a farmer to adopt SPS at a medium–high adopter category (A4).

**Figure 2:**
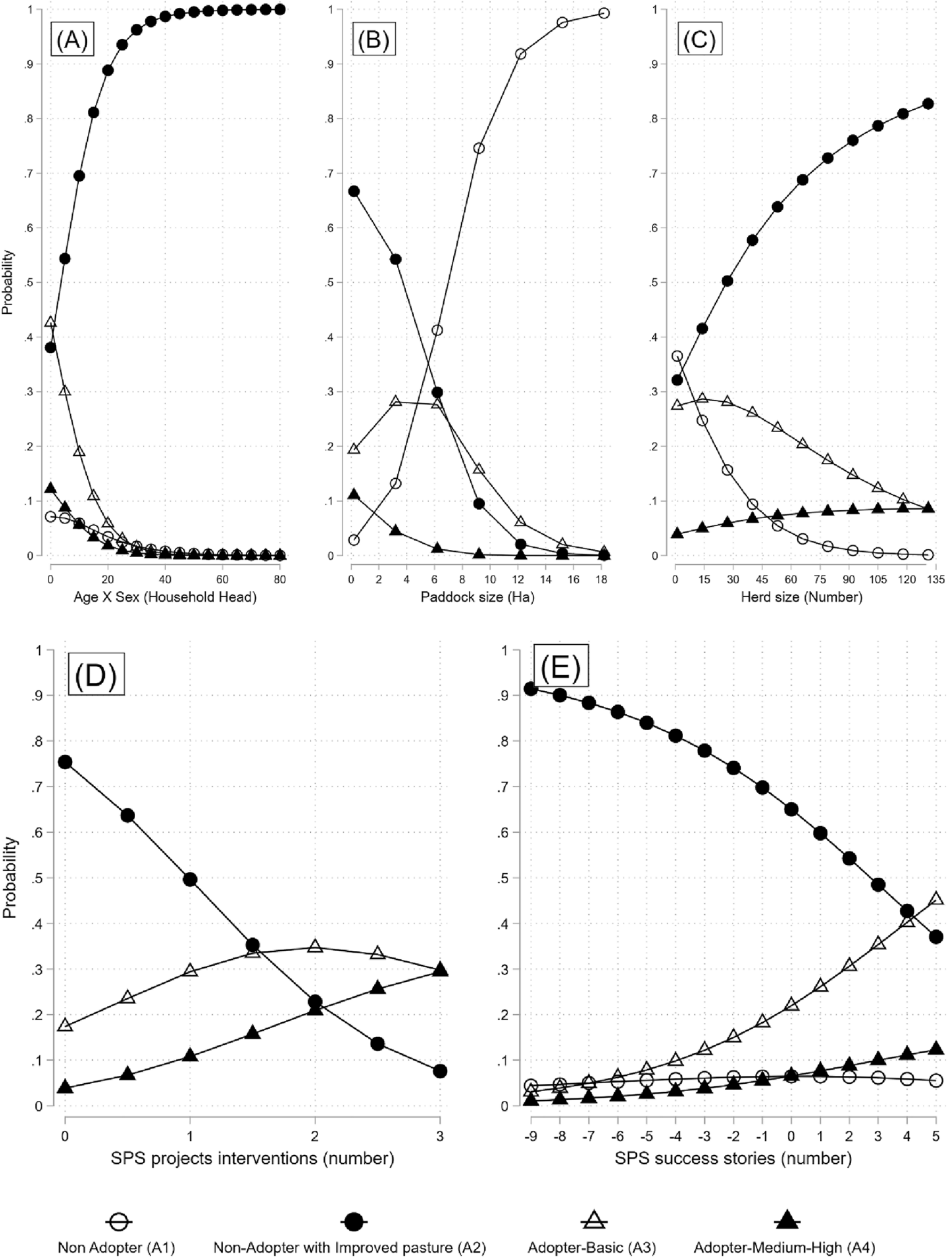
The effect of socioeconomic factors on the probability of being classified in defined paddocks SPS categories adoption. (**A**) The effect of age and gender of household interaction on the predicted probability of being classified in SPS adoption levels. (**B**) The effect of paddock size on the predicted probability of being classified in SPS adoption levels. (**C**) The effect of total herd size on the predicted probability of being classified in SPS adoption levels. (**D**) The effect of the number of SPS projects for which the farm has been intervened on the predicted probability of being classified in SPS adoption levels. (**E**) The effect of the number of SPS success stories that the household head knows (municipality vs. Others) on the predicted probability of being classified in SPS adoption levels.

The analysis in this study further revealed a distinctive non-linear relationship between SPS adoption and socioeconomic indicators. Notably, paddocks with larger dimensions exhibit a greater propensity to fall within the non-adopter category (Fig. 2B). This observation suggests the prevalent practice of extensive ranching in the study area, characterized by utilizing extensive land areas with a relatively low number of paddocks. In contrast, the adoption of sustainable silvopastoral systems demands cattle farmers to partition paddocks, allowing for the implementation of diverse management strategies and the reduction of individual paddock sizes^35^.

Farms with a higher number of cattle and an older female head of household are more likely to have a paddock classified as non-adoptive with improved pasture (Fig. 2A–C). On the other hand, participating in more projects, events, and training related to SPS increases the probability of paddocks being classified as adopters (Fig. 2D). The number of SPS projects and number of SPS success stories in the area increases the likelihood that a paddock is classified as adopter basic or medium–high and reduces the likelihood that a paddock is classified as non-adopter with improved pasture (Fig. 2E). These results further show that obtaining knowledge and inputs on SPS is important in adopting sustainable technologies (A3 and A4) at the paddock level (Fig. 2D). However, knowing the technology is not enough to adopt it at a higher intensity; Fig. 2 E shows that if the farmer knows more cases of SPS success in his municipality than in others, he has a higher probability of adopting a basic level in his paddocks (from 0.05 to 0.45). Still, the likelihood that a paddock is classified as a medium or high adopter is lower (from 0 to 0.10).

## Discussion

Through an examination of intra-farm level land use decisions, our research provides new insights into the factors influencing the adoption of SPS and sheds light on the challenges and opportunities for scaling these practices. This paper’s findings hold policy implications for the scaling of low-emissions livestock production systems in the Colombian Amazon and elsewhere. Our results highlight the importance of: (1) promoting paddock division and implement diverse management strategies to facilitate transition towards SPS adoption; (2) reaching out to farmers who have not yet participated in SPS projects and enabling invests in low-emissions livestock production; (3) targeting female heads of household in scaling efforts, (4) recognizing and communicating the broader benefits of SPS beyond production and financial benefits.

However, it appears that the income generated from the sales of milk and beef is higher in non-adopter levels where improved pasture is present, this results were in line with those of reported by Lee et al. This could be due to various environmental factors and the possibility that adopters have not fully implemented the SPS on their farms. Hence, farms that adopt SPS may not immediately perceive or realize the full spectrum of potential economic benefits associated with this technology, as these benefits tend to become more evident in the medium and long term. Although the differences in income are not significant, it indicates that the implementation of SPS systems is a gradual process and that once fully implemented, income levels are expected to increase significantly^36,37^. Therefore, the successful implementation of SPS systems is expected to greatly enhance the quality and quantity of milk and beef production.

Results from this study are consistent with previous studies reporting that SPS adoption is a gradual process that requires time, information, and inputs for testing and implementation^14,38^ and highlight the need for targeted interventions and support to facilitate the transition to sustainable practices. This research diverges from conventional farm-level analysis and adopt a focused approach that centers on paddocks as the primary unit of analysis to examine the determinants of adoption of Silvopastoral Systems (SPS). This study also delves into the farm-level benefits associated with SPS adoption, including improved productivity, increased income, and the potential for land conservation. Results from this study further indicate that some of the surveyed farmers have taken steps towards adopting sustainable practices but have not fully transitioned to adoption of SPS. This is probably because of socio-economic factors that act as barriers to the full adoption of SPS^20^. Which in turn suggests that there is potential to further enhance SPS adoption among these farmers. However, the percentage adoption in this area is low which can be explained by the ageing farming population of this area dominated by male farmers. Specifically, results show that only a small proportion (5%) of paddocks exhibit a high or medium level of SPS adoption across all their paddocks. Similarly, results reveal that a significant majority of paddocks (76%) of the surveyed farmers are not classified as SPS adopters. Results also revealed that the highest proportion of paddocks falls into the category non-adopter with improved pasture category (42%). This suggests that there may be economic and environmental incentives for adopting rotational grazing, which could explain why the non-adopter group with improved pasture category had the largest proportion^39^. However, there may be other factors beyond economic incentives that prevent some farmers from adopting SPS practices. These could include lack of information or knowledge about the benefits of SPS^26^, cultural resistance to change^27^, or difficulty in implementing the practice due to technical or logistical challenges^39^.

This results further emphasize the importance of considering farm and farmer level characteristics and engaging farmers in the adoption process. The successful scaling of low-emissions livestock production systems relies on tailoring interventions to the specific needs and capacities of different farms and farmers^40^. This is consistent with several studies highlighting the importance of customization in promoting sustainable practices and ensuring widespread adoption^31,41,42^. That is, variation in farm size, herd size and size of paddocks across farms are critical variables when scaling low-emissions livestock production. For instance, small-scale farmers may require different support mechanisms compared to large-scale farmers. Meanwhile, managing livestock across different herd sizes presents unique challenges. Example, small-scale producers may face difficulties in accessing markets and veterinary services, hindering their productivity and profitability, while, large-scale producers may require assistance in implementing sustainable intensification practices to mitigate environmental impacts associated with larger herds^43–45^.

Policies and assistance programs should recognize these variations and provide targeted support based on farm sizes, ensuring that all farmers receive relevant guidance and resources. Similarly, the age of the household head is another critical variable, in our sample the average age of the household head is 52 years, indicating the need for intergenerational knowledge transfer and capacity building. Therefore, to ensure the continuity of sustainable livestock production practices, it is crucial to encourage younger generations to adopt and innovate sustainable farming techniques while leveraging the traditional knowledge and experience of older farmers^46,47^. This intergenerational collaboration would foster the transfer of knowledge and skills necessary for long-term sustainability.

### Promoting paddock division and implementing diverse management strategies to facilitate transition towards SPS adoption

The variation in the number and size of paddocks across farms presents opportunities for optimizing paddock management and resource utilization. The average number of cattle paddocks on a farm (27) implies that farmers manage multiple paddocks for grazing purposes. Meanwhile, the results of the econometric models indicate that an increase in paddock size increases the probability that a paddock will be classified as non- adopter. This observation implies that the probability of adopting SPS practices at the paddock level increases with smaller paddocks, while simultaneously having a greater number of paddocks on the farm. This result is similar to the results of Teague and Barnes^48^ who found that the frequency of rotational grazing was influenced by farm size, the number of available paddocks, and whether the farm was primarily for livestock or crops^48^. They also found that cost and labor constraints were major barriers to adoption.

Moreover, the finding that non-adoption at the paddock level is positively associated with the paddock's size suggests the prevalence of extensive livestock practices in the study area. This implies that extensive ranching practices may contribute to lower adoption of sustainable grazing practices, which could have negative impacts on soil health, ecosystem functioning, and livestock productivity. This indicates that promoting paddock division and implementing diversified management practices, such as improved pastures, can facilitate the adoption of sustainable practices. Through targeted training programs and extension services, farmers can learn and adopt best practices for paddock rotation. Implementing efficient paddock rotation strategies, improves forage quality, reduces soil erosion, and enhances livestock health and productivity^49^. Therefore, policy interventions aimed at encouraging smaller paddock sizes and promoting sustainable land use practices can contribute to scaling up low-emissions livestock production systems.

### Recognizing and communicating the broader benefits of SPS

The results of this study suggest that while Category_Farm-FA1 might have higher stocking rates and calving rate variability, the adoption of specific practices in Category_Farm-FA4 has led to relatively consistent calving rates and income stability, despite having fewer farms. This may be attributed to more effective management practices or strategies that have resulted in better income from milk and animal sales, even when the sample size is smaller. However, it is important to consider other factors that may have influenced these results, such as variations in market conditions, production practices^50^, or other economic factors. Broader benefits of SPS, include improved soil health, biodiversity conservation, and climate resilience^51,52^, which contribute to the sustainable development of the Colombian Amazon region.

The results of this study suggest that the adoption of SPS is not a sudden or immediate process, but rather a gradual and long-term trajectory. This implies that farmers and agricultural practitioners need to be patient and persistent in their efforts to implement sustainable practices. It is not enough to simply provide them with information and resources; they also require sufficient time for on-farm experimentation and learning. This finding further emphasizes the need for continuous support and guidance throughout the adoption process. Also, the adoption of SPS requires a steadfast commitment to conservation practices^53^. It is not a one-time action, but an ongoing commitment to sustainable and environmentally friendly practices. This commitment involves not only the initial implementation of SPS but also the maintenance and continuous improvement of these practices over time. It requires a mindset shift towards prioritizing long-term sustainability over short-term gains.

However, SPS adoption is not only beneficial for farm sustainability and productivity but is also associated with a higher commitment to conservation initiatives. This can have several positive effects on efforts to achieving the following Sustainable Development Goals (SDGs: no poverty (SDG1); gender equality (SDG 5); climate action (SDG 13); and Life on land (SDG 15). It indicates that the adoption of sustainable practices on farms goes hand in hand with a heightened sense of responsibility and participation in broader conservation efforts, highlighting the potential for a win–win scenario where agriculture and environmental conservation can mutually benefit^54^.

Although the immediate financial benefits may not be apparent from SPS adoption, policymakers should consider and communicate the long-term ecological and socio-economic advantages. Furthermore, the absence of statistically significant positive differences in income from milk sales and from sales of animals among farms with different levels of SPS adoption is an important result to consider. While financial gains associated with SPS adoption are long term, it is crucial to acknowledge the potential long-term benefits that SPS practices can bring, such as enhanced ecosystem services, improved soil fertility, and resilience to climate change. These non-monetary benefits, although not captured directly in the income indicators, contribute to the overall sustainability and resilience of the livestock production systems.

Significant variations in certain cattle production indicators exist among farms according to their degree of SPS adoption. Notably, there were substantial positive variations in stocking rates among farms that varied in their degree of SPS adoption within their paddocks. This enhancement of stocking rate levels implies that SPS practices possess the potential to augment the overall productivity of cattle farms in the long term. This finding aligns with the goals of low-emissions livestock production systems, as it suggests that SPS adoption does not lead to a trade-off between environmental sustainability and productivity.

Moreover, our results also suggest that farms that comprised a combination of paddocks with natural and improved pastures, planted and stable trees, livestock aqueducts, and fodder banks exhibited a higher percentage of calving cows. This finding suggests a positive relationship between the adoption of diverse SPS components and calving rates, potentially indicating improved reproductive performance and herd health in such farms. These results are consistent to previous studies and highlight the importance to put in place environmental safeguards to prevent deforestation leakage from efforts to scaling SPS^15^.

### Reaching out to farmers who have not yet participated in SPS projects and enabling investments in low-emissions livestock production

Participation in SPS projects does not necessarily translate into immediate adoption of SPS. Results from this study indicate that farmers have on average benefited from SPS projects at least 0.64 times. However, the high standard deviation suggests a concentration of project resources on some farmers, leaving others with limited access to support. This finding further suggests that some non-adopter farmers engage in SPS projects as a means of exploring alternative approaches or seeking support for their existing practices^55^. Widespread adoption of low-emissions livestock production systems demands an equitable distribution of projects and resources^56^.

Therefore, policymakers and project implementers should prioritize reaching out to farmers who have not yet participated in SPS projects, ensuring that they receive necessary resources, training, and technical assistance while making available incentives for incentivizing farmers who already participate in SPS projects. This approach fosters a sense of inclusivity and ensures that the benefits of sustainable livestock production reach all farmers in the region, regardless of their previous project participation. Particularly, considering that our model results suggest that the proximity of positive SPS interventions has a significant impact on the likelihood of adopting higher SPS levels in paddocks. Additionally, paddocks that participate in multiple projects, events, and training related to SPS are more likely to be classified as SPS adopters. This, in turn, reduces the probability of a paddock being a non-adopter with improved pasture^57^. To address this, policy interventions should focus on providing targeted training and technical support to farmers who are non-adopters, ensuring that they have the necessary resources and knowledge to transition towards sustainable practices^41^.

Furthermore, the negative association of high medium adoption with age could be explained by the fact that, older household heads may be more resistant to change and less likely to adopt new agricultural practices, including the silvopastoral system. They may have established traditional farming methods and may be less inclined to adopt innovative approaches. Additionally, our results support previous studies that conclude that addressing credit constraints is essential to facilitate the adoption of sustainable practices by providing farmers with the financial resources necessary for their implementation^58^, Policymakers, financial institutions, and stakeholders should collaborate to improve access to credit for farmers, enabling them to invest in low-emissions livestock production technologies and infrastructure^59^. In particular, our results indicate that 56% of the surveyed farmers have access to credit. They also indicate that the variable access to credits is positively linked with both non-adopter with improved pastures and medium–high adopter paddocks but negatively associated with basic adopter paddocks. This underscores the notion that when a non-adopter paddock starts incorporating pastures, it necessitates a financial investment for land preparation and division. Similarly, transitioning from being a basic adopter to a more intensive stage of adoption involving elements like livestock agreements and live fences also requires augmented financial support. Despite this we argue that further analyses are needed to explore credit access and its implications for the scalability of these production systems.

### Targeting female heads of household

Targeting female heads of household can be an effective approach for scaling sustainable livestock production systems. Policymakers should consider gender-specific interventions, such as providing training, financial resources, and support networks for female-headed households to enhance their capacity to adopt and implement SPS practices^60^. It is evident that most farmers are male, with women representing only 15% of the sampled farmers. This gender disparity is a crucial factor to consider when designing interventions and policies to promote sustainable livestock production moreover our multinomial logit results shows that female farmers are more likely to adopt SPS practices compared to their male counterparts^20^.

By targeting female heads of household, we can empower women and enhance their participation in decision-making processes related to livestock production. This can lead to more inclusive and sustainable practices on farms. Furthermore, the findings highlight the importance of gender in SPS adoption. Female-headed cattle farms with fewer paddocks have a lower probability of being non-adopters and are more likely to adopt SPS practices at the paddock level. It is observed in our results that, on average, female heads of households are more motivated to conserve forests on their farm for environmental reasons than their male counterparts (see Appendix A.3). Additionally, even though the differential between the economic value of the land on their farm and that of their neighbors is greater for male heads of households (Approx. 1 million $COP) than for females (Approx. 0.5 million $COP), female heads of households consider that this difference is because, on their farm, there is the presence of water sources and biodiversity. These results suggest that women adopt SPS more intensively because they are more willing to conserve and protect the environment than men. As other studies have reported, women often play a significant role in agricultural activities, including livestock management, but their contributions are often undervalued and overlooked^61^.

In a nutshell, our findings indicate that the inclusion of edible shrubs and trees in SPS significantly enhances their sustainability and economic viability. This is consistent with other successful examples from papers already listed above, where such systems have led to improved farm productivity and environmental benefits. The use of these plants not only supports livestock nutrition, thereby improving productivity and health but also contributes to better carbon sequestration and biodiversity, compared to traditional grazing systems. However, despite these benefits, our study shows that adoption rates are still low, largely due to the complexity of SPS management and initial establishment costs. Promoting awareness and providing targeted support for the implementation of SPS that includes edible trees and shrubs can address these barriers.

## Conclusion

This study provides valuable insights into the factors influencing the adoption of SPS at paddock levels thus shedding more light on the challenges and opportunities for scaling these practices in low-emissions livestock production systems. This study further underscores the importance of integrating edible shrubs and trees into SPS as a strategy for sustainable livestock production. Such integration not only supports the ecological and economic goals of SPS but also promotes broader environmental conservation efforts. Our results complement the growing body of literature on the farm level adoption of SPS by focusing on benefits of SPS adoption and breaking even in investigating adoption at paddock level rather than farm level as previous research has concentrated on. The results of this study show that conveying information about the technology and its potential benefits boosts SPS adoption. Additionally, results from this research demonstrate that SPS adoption does not lead to farm level trade-offs between environmental sustainability and productivity. However, it highlights the importance of tailoring interventions to the specific needs and capacities of different farms and farmers' characteristics, promoting paddock division, providing targeted training and technical support to farmers who are non-adopters, targeting female heads of household, and recognizing and communicating the broader benefits of SPS beyond production and financial benefits. Further research should, therefore, explore the specific types of edible plants most beneficial for inclusion in SPS across different ecological zones to tailor recommendations that maximize both environmental and production benefits. Future research should also further delve into the environmental, productive, and economic variables that require consideration by strategies aiming to scale SPS practices.

### Supplementary Information

Supplementary Information.

## Data Availability

The data used for this study is readily available upon reasonable request. This request can be done by contacting the corresponding author through augusto.castro@cgiar.org.
