# Supplementary material for: Unlocking sustainable livestock production potential in the Colombian Amazon through paddock division and gender inclusivity
Source: Sci Rep. 2024 Jun 13;14:13644. doi: 10.1038/s41598-024-63697-2 (PMC11176341; doi:10.1038/s41598-024-63697-2)
Supplement: Supplementary file 1 — Supplementary Information. [file 41598_2024_63697_MOESM1_ESM.docx]

**Supplementary Materials**


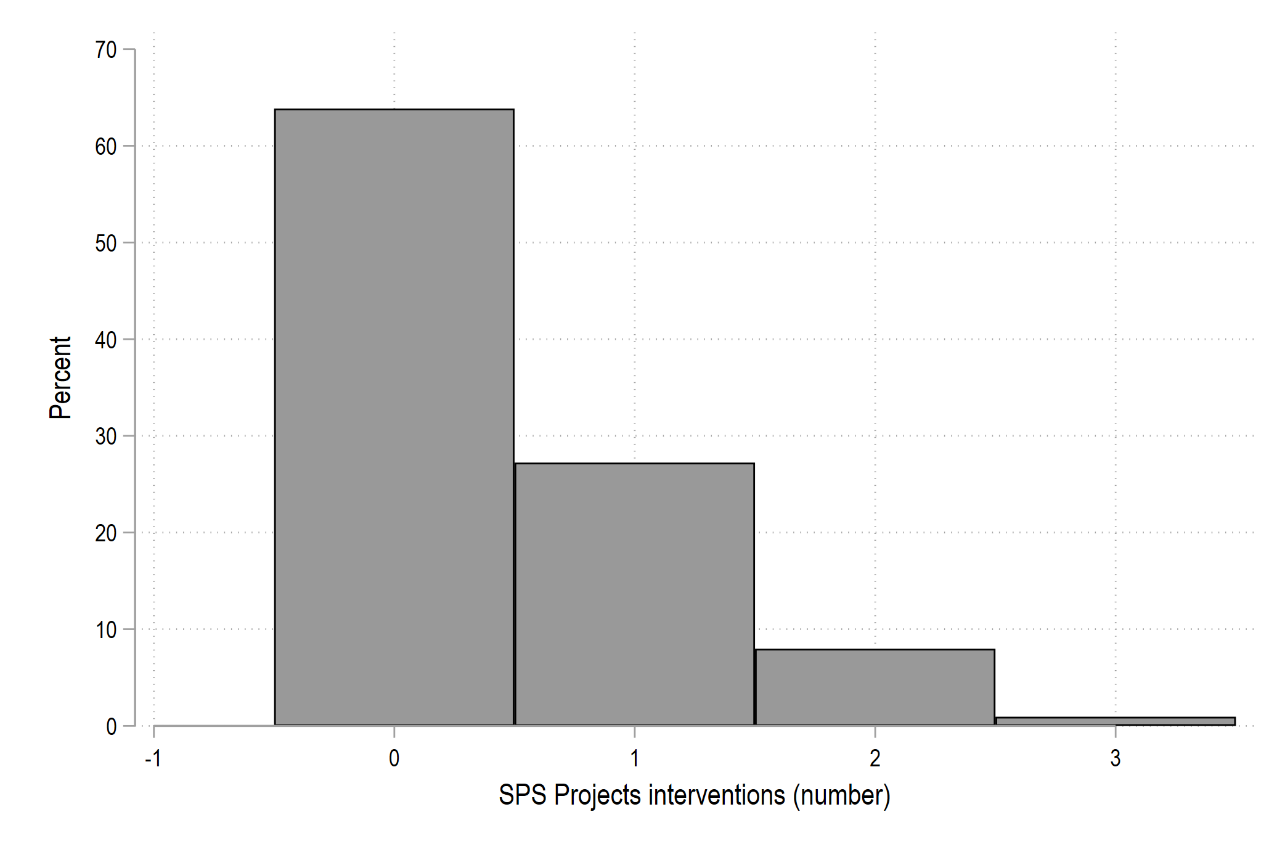


**Supplementary figure 1 Histogram of SPS Projects interventions (number).**

**Supplementary Table 1. Indicators of Cattle Productivity Based on Paddock-Level SPS Adoption across Farm SPS Categories.**

|  | N paddocks | Mean | S.D. | Comparation | Mean Difference | Std. Error | T-test-pvalue ^α^ |
| --- | --- | --- | --- | --- | --- | --- | --- |
| Stocking rate (AU/Ha) | | | | | | | |
| Farm-FA1 | 237 | 1,21 | 1,30 |  |  |  |  |
| Farm-FA2 | 1549 | 1,07 | 0,60 | FA2 vs FA1 | -0,15* | 0,05 | 0,03 |
| Farm-FA3 | 732 | 1,18 | 0,83 | FA3 vs FA1 | -0,04 | 0,06 | 1,00 |
|  |  |  |  | FA3 vs FA2 | 0,11* | 0,34 | 0,01 |
| Farm-FA4 | 230 | 1,40 | 0,82 | FA4 vs FA1 | 0,19* | 0,07 | 0,05 |
|  |  |  |  | FA4 vs FA2 | 0,22*** | 0,06 | 0,00 |
|  |  |  |  | FA4 vs FA3 | 0,33*** | 0,05 | 0,00 |
| Calving rate (%) | | | | | | | |
| Farm-FA1 | 206 | 54,63 | 31,34 |  |  |  |  |
| Farm-FA2 | 1552 | 61,94 | 16,93 | FA2 vs FA1 | 7,31*** | 1,47 | 0,00 |
| Farm-FA3 | 745 | 67,54 | 22,19 | FA3 vs FA1 | 12,92*** | 1,56 | 0,00 |
|  |  |  |  | FA3 vs FA2 | 5,61*** | 0,88 | 0,00 |
| Farm-FA4 | 230 | 68,11 | 15,74 | FA4 vs FA1 | 13,48*** | 1,90 | 0,00 |
|  |  |  |  | FA4 vs FA2 | 6,17*** | 1,40 | 0,00 |
|  |  |  |  | FA4 vs FA3 | 0,57 | 1,49 | 1,00 |
| Milk productivity (Lt/cow/day) | | | | | | | |
| Farm-FA1 | 133 | 3,71 | 1,92 |  |  |  |  |
| Farm-FA2 | 1519 | 4,66 | 3,12 | FA2 vs FA1 | 0,96*** | 0,24 | 0,00 |
| Farm-FA3 | 718 | 4,00 | 1,83 | FA3 vs FA1 | 0,30 | 0,25 | 1,00 |
|  |  |  |  | FA3 vs FA2 | -0,66*** | 0,12 | 0,00 |
| Farm-FA4 | 228 | 3,35 | 1,54 | FA4 vs FA1 | -0,36 | 0,29 | 1,00 |
|  |  |  |  | FA4 vs FA2 | -1,32*** | 0,19 | 0,00 |
|  |  |  |  | FA4 vs FA3 | -0,65* | 0,20 | 0,01 |
| Income from sales of animals (million COP/yr) | | | | | | | |
| Farm-FA1 | 38 | 2,06 | 0,99 |  |  |  |  |
| Farm-FA2 | 620 | 8,41 | 10,13 | FA2 vs FA1 | 6,36*** | 1,40 | 0,00 |
| Farm-FA3 | 262 | 3,08 | 5,67 | FA3 vs FA1 | 1,02 | 1,46 | 1,00 |
|  |  |  |  | FA3 vs FA2 | -5,34*** | 0,25 | 0,00 |
| Farm-FA4 | 151 | 4,39 | 5,73 | FA4 vs FA1 | 2,33 | 1,52 | 0,76 |
|  |  |  |  | FA4 vs FA2 | -4,03*** | 0,77 | 0,00 |
|  |  |  |  | FA4 vs FA3 | 1,31 | 0,87 | 0,79 |
| Income from milk sales (million COP/yr) | | | | | | | |
| Farm-FA1 | 130 | 10,71 | 5,16 |  |  |  |  |
| Farm-FA2 | 1483 | 25,57 | 26,83 | FA2 vs FA1 | 14,86*** | 1,97 | 0,00 |
| Farm-FA3 | 701 | 19,71 | 11,56 | FA3 vs FA1 | 9*** | 2,06 | 0,00 |
|  |  |  |  | FA3 vs FA2 | -5,86*** | 0,99 | 0,00 |
| Farm-FA4 | 206 | 15,65 | 6,76 | FA4 vs FA1 | 4,95 | 2,42 | 0,25 |
|  |  |  |  | FA4 vs FA2 | -9,91*** | 1,61 | 0,00 |
|  |  |  |  | FA4 vs FA3 | -4,05 | 1,71 | 0,11 |

**Note: *** p<0.01, ** p<0.05, * p<0.1. ^α^ Adjustment for multiple comparisons Bonferroni.**

**Supplementary Table 2. Conservation motivations and land price differential by gender of the head of household**

| **Variables** | **Sex of household head** | | | | | |
| --- | --- | --- | --- | --- | --- | --- |
|  | **Male** |  | **Female** |  | **Difference** |  |
|  | **mean (1)** | **sd (2)** | **mean (3)** | **sd (4)** | **(3-1)** | **T-test-pvalue** |
| **The main reason why they keep the forest on their farm ( Dummy variables)** |  |  |  |  |  |  |
| A) It belongs to a conservation agreement | 0.73 | 0.45 | 0.71 | 0.46 | -0.02 | (0.837) |
| B) To protect the water source | 0.46 | 0.50 | 0.45 | 0.51 | -0.01 | (0.956) |
| C) To have shade/coolness (humans) | 0.67 | 0.47 | 0.61 | 0.50 | -0.06 | (0.551) |
| D) To have shade/coolness (animals) | 0.01 | 0.08 | 0.00 | 0.00 | -0.01 | (0.319) |
| E) To hunt | 0.03 | 0.17 | 0.00 | 0.00 | -0.03 | (0.045) |
| F) To collect fruits | 0.11 | 0.31 | 0.06 | 0.25 | -0.04 | (0.416) |
| G) To capture subsidies | 0.11 | 0.31 | 0.06 | 0.25 | -0.04 | (0.416) |
| H) To reserve money (timber,animals) | 0.35 | 0.48 | 0.45 | 0.51 | 0.10 | (0.313) |
| I) As a reserve for construction | 0.95 | 0.22 | 0.84 | 0.37 | -0.11* | (0.119) |
| J) To protect the environment | 0.44 | 0.50 | 0.58 | 0.50 | 0.14* | (0.152) |
| K) To pass it on to children/grandchildren | 0.04 | 0.19 | 0.03 | 0.18 | 0.00 | (0.924) |
| **How much does the land value $ COP/(ha)?** |  |  |  |  |  |  |
| a) In the area/village ( $ Millions/Ha) | 6.69 | 9.04 | 7.72 | 11.03 | 1.03 | (0.604) |
| b) On his farm ( $ Millions/Ha) | 7.72 | 9.80 | 7.98 | 10.80 | 0.26 | (0.895) |
| Difference (b-a) | 1.02 | 5.94 | 0.42 | 1.64 | -0.61 | (0.277) |
| **Main reasons to explain the price differential ( Dummy variables)** |  |  |  |  |  |  |
| Proximity to access roads | 0.69 | 0.46 | 0.64 | 0.50 | -0.06 | (0.730) |
| Presence of water sources | 0.55 | 0.50 | 0.64 | 0.50 | 0.09 | (0.591) |
| Presence of biodiversity | 0.39 | 0.49 | 0.55 | 0.52 | 0.16 | (0.360) |
| land quality | 0.45 | 0.50 | 0.45 | 0.52 | 0.00 | (0.994) |
| Implementation of the silvopastoral system | 0.51 | 0.50 | 0.55 | 0.52 | 0.04 | (0.821) |
| Observations | 160 |  | 40 |  | 200 |  |
